# A Citation Analysis of the Top 50 Most Cited Nurse Practitioner Publications

**DOI:** 10.7759/cureus.74406

**Published:** 2024-11-25

**Authors:** Alana Halfpenny, James S Huntley

**Affiliations:** 1 Nursing, Queen's University, Kingston, CAN; 2 Orthopedic Surgery, Primary Children's Hospital, Salt Lake City, USA

**Keywords:** 50 most cited, advanced practice nurse, advance practitioner nurse, bibliometrics analysis, citation analysis, classic article, nurse practitioner (np)

## Abstract

The role of nurse practitioners (NPs) has become integral to healthcare systems worldwide. Originating in the United States over 50 years ago, it has since been adopted by countries such as Canada, the United Kingdom, and Australia. To honor the research and contributions that have shaped the NP discipline, it is valuable to review and recognize key literature that has significantly impacted its development. Bibliometrics, a research methodology, offers an objective lens for evaluating the influence of scholarly articles on the evolution of a discipline. Citation analysis (CA), a key method in bibliometrics, examines how frequently a publication is cited by others, often serving as a measure of its impact, influence, and contribution to its field. This study aims to identify the top 50 most cited publications related to NPs in the Web of Science (WoS) database to review and describe the influential works that have contributed to the profession’s growth. Comparisons are drawn with a parallel review in Scopus and recent NP-related bibliometric studies. In 2021, a structured search was conducted using the WoS Core Collection with key terms such as “Nurse Practitioner” and “Advanced Practice Nurse”. Inclusion and exclusion criteria were applied, and publications were ranked by citation count from highest to lowest. The analysis covered citation counts, topics, publication dates/types, country of origin, author details (institution and discipline), and journal characteristics (e.g., impact factor, IF). The top 50 most cited articles and their characteristics are presented. Citation counts ranged from 78 to 656, with publication dates spanning six decades across 30 journals, 38 institutions, and 194 authors. The leading authors were Mary O’Neil Mundinger, Denise Bryant-Lukosius, and Alba DiCenso. Topics included the role’s impact and development, registration/licensing, and scope of practice. Most articles (n = 35) were published in journals with an IF greater than 2. This review offers a systematic approach to identifying seminal works that have influenced the NP profession globally. While CA has its limitations, it provides a valuable method for literature review. This study contributes valuable insights into the history and development of NP research and offers guidance for future research efforts.

## Introduction and background

The nurse practitioner (NP) role has become a common entity in healthcare organizations around the world. The development, acceptance, and integration of this role is in various stages internationally but had its inception in the United States [1]. The work of Drs Silver and Ford formally pioneered the concept in the late 1960s; however, nurses in advanced roles, such as midwives and nurse anesthetists, were evident as early as the 1940s [2]. Since then, the NP role has been adopted beyond the United States, notably in Canada, Latin America, Asia, Australia, New Zealand, and the United Kingdom [1,2].

Given the longevity and pervasiveness of the NP profession, we wished to consider and identify the sentinel underlying literature and its contributions to the “modern-day” NP. As a profession evolves, the increased volume of associated research itself demands such a description and preliminary analysis [3]. We sought to explore the literature using a bibliometric approach.

Bibliometrics

Bibliometrics is a research methodology using a variety of statistical and mathematical tools to explore the characteristics of publications such as books or journal papers [4]. Bibliometric techniques can provide an objective reflection on the possible influence of scholarly articles on the maturation of a discipline [5-7] and further garner an appreciation of the advances and contributions in a particular field [8]. Citation analysis (CA), as a key method in bibliometrics, identifies how often an academic publication is referenced or cited [9,10]. CA is frequently considered to provide a measure of the strength, or importance of a publication - its impact, influence, and value contribution to its field [5,10-14]. Using metrics in CA, one can assess the present state of research, reflect on seminal works, and identify the most productive researchers, organizations, and countries, as well as highly influential journals [9,15,16]. For example, the use of an impact factor (IF) in a journal for which an article is published can have some perceived significance. The numerical rating in the form of an IF of a journal is used as a proxy surrogate for the quality or influence and is used to rank journals using elements of citation patterning over a designated timeframe [17,18].

Bibliometric studies: NP

Bibliometric research has been used regularly in medicine, and there is an increase in its use in nursing [4]. Several studies have used bibliometrics focusing on NPs: Currie et al. [19] highlighted primary Australian NP studies using bibliometric techniques to identify possibilities for future research; Waldrop et al. explored publication metrics of highly cited NP-related articles in the Journal for Nurse Practitioners to assess the impact of the journal and its reach [20]; Huang et al. explored NP publications using bibliometrics and visualization of the research status using CiteSpaceV, downloading the studies in June 2019 [21]; Jennings and Tori [22] conducted a bibliometric analysis in September 2021 of the top 100 NP-related publications, using the database Scopus. Their study covered publications published from 2007 to 2021 to reflect contemporaneous NP literature.

## Review

Aim 

The aim of this study is to identify the top 50 most frequently cited publications related to NPs in the Web of Science (WoS) and to describe the sentinel written work contributing to the evolution of the profession. Despite using different databases, time periods of publications, and different search strategy criteria, we also aim to highlight the top publications from our study based on citation frequency, compared with our concurrent Scopus search and the studies by Huang et al. and Jennings and Tori [21,22].

Methods 

Our methods were analogous to previous protocols and methodologies of CA [3,5-8,12-14,23,24]. The process for a bibliometric study requires transparency to be faithfully reproducible [15]. The specifics of how the dataset analyses were obtained are detailed.

Data Collection Tool

Different databases are used with CA, such as WoS, Scopus, and Google Scholar [25]. We chose WoS as it is considered a trusted resource in bibliometrics, the “most authoritative and influential academic journals” [6,26], and has access to publications dated back to the 1900s [27] and a clear method of tracking publication citation data, with a distinct allocation of disciplines using categories [5]. WoS offers access to numerous publications in core journals, conference proceedings, and datasets related to medicine, nursing, engineering, the social sciences, and humanities [3].

We used the Journal Citation Report (JCR) database for the determination of the journal IF as well as the category of a particular journal. The JCR is a yearly report on the citation impact of journals at a given moment in time. The IFs for the journals identified in our final 50 publications were retrieved on February 27, 2021.

Search Strategy

Structured searches of the WoS Core Collection were completed on January 30, 2021, resulting in the final 50 publications. The time span for the search was years 1900 to 2020, all languages and all six citation indexes. We intentionally chose the time span to access papers that were possibly influential historically - in short, sentinel papers. Details on the specific search strategy are available upon request. The use of both “Nurse Practitioner” and “Advanced Practice Nurse” (APN) was intentional in our search strategy to compensate for past use of the terms used interchangeably; term use is also influenced by the country of origin [28]. There is improved clarity and distinction around the roles of NPs and APNs [1], but we wanted to ensure past literature that used either term was captured.

The results of this search strategy yielded 6,081 records. Using WoS, these records were sorted by “times cited, highest to lowest.” The records were then reviewed manually by both investigators to determine suitability for our dataset of 50, according to inclusion and exclusion criteria.

Inclusion and Exclusion Criteria

The inclusion criteria required evidence of direct and meaningful discussion, investigation, or focus on the NP/APN domain. The initial top 50 highly cited publications were screened by review of the “title”, then the “abstract” review, and followed by a final appraisal by reading the publication to determine its suitability. It was intended that if any discrepancies arose regarding publication inclusion and exclusion, a discussion would occur between the investigators; however, no discrepancies arose.

The first screen involved a title review to determine whether it was relevant to our area of inquiry. If the title was vague, or if it met our initial “title screen,” a review of the abstract was conducted. If both the title and abstract were still not clear, then the publication was reviewed in its entirety. If a publication was excluded, the next publication was added to the list and reviewed, based on descending citations. Once screened and accepted for full analysis, the top 50 records comprising our final data set were marked, ranked according to a number of citations, and saved within the WoS database, as well as exported to Microsoft Excel (Microsoft Corporation, Redmond, WA, United States) and Microsoft Word (Microsoft Corporation).

Areas Examined

In addition to reviewing citation counts that dictated ranking, we also examined topics, publication dates, publication types/research design, country of origin, institutions first-authors, author discipline, and journal characteristics. We also performed a similar search in the Scopus database. For clarity, the next section details an explanation regarding the topic definition and our Scopus search.

Topic

Four topic areas were developed into which each of the 50 publications could then be assigned. Topic development arose from the subject matter derived from the article itself within the data set. A thorough review of each article was conducted by AH with the topics clearly delineated and then further discussion and consensus between the investigators of what each topic area meant. We recognized that a publication may fall into one or more of our topic areas and strived to identify when a substantial overlap of topic areas occurred. There were no discrepancies in assigning the topic area to a publication between the investigators. The evolution of our topic categories was influenced by the work of Benton et al. [15]. We developed four main areas of topics: scope of practice, regulation/licensing, role development, and impact of role.

We defined the four topic areas as follows: Scope of practice is the professional services provided by NPs that he/she is permitted to do as sanctioned by a governing body. This description is supported by Kleinpell et al. [29], who describe the scope of practice as the activities that are permitted for a healthcare practitioner. Regulation/licensing involved discussions related to aspects of governing bodies, laws, and legislative actions for NPs to provide their services in a safe and competent manner. Role development revolved around implementation, nomenclature, progression, growth, integration, and education of the NP role. Impact of the role focused on patient satisfaction, health care outcomes, costs, and/or economics and safety.

Once agreement was reached on what each topic area meant/covered, one investigator (AH) assigned each publication to one of the four topic areas. Then both investigators reviewed the assigned topics for each publication, discussed, and agreed that the topic was accurate for each publication.

Scopus Search: Comparison

For comparison, a similar search was completed in Scopus on January 18, 2021. Scopus data includes peer-reviewed titles from international publishers, open access journals, conference proceedings, and trade publications. Title field and the same search terms: nurse practitioner OR advanced practice nurse yielded 6,530 results. The same inclusion and exclusion criteria were applied.

Results

Citation Frequency

The 50 most-cited publications and characteristics are shown in Table 1 [2,30-78]. The number of citations per article ranged from 78 to 656 (mean: 149; median: 107). The most cited paper was “Systematic review of whether nurse practitioners working in primary care can provide equivalent care to doctors” authored by Horrocks et al., published in the BMJ [30]. It received 656 citations over 19 years, with the first citation by Rifkin et al. [79] and the most recent by Bezze et al. [80]. This paper was ranked as the third highest citation frequency (57) by Huang et al. [21], with the “most strength burst” indicating a significant and rapid increase in the number of citations received. Overall, 14 articles in our top 50 had accumulated 150 or more citations.

**Table 1:** Top 50 most cited NP publications ^*^ Changed to Rheumatology ^**^ Changed to Journal of the American Association of Nurse Practitioners ^***^ Changed to Journal of Trauma and Acute Care Surgery IF, impact factor; NP, nurse practitioner

Rank	Citations	First author	Title	Journal	Year	IF
1	656	Horrocks S [30]	Systematic review of whether nurse practitioners working in primary care can provide equivalent care to doctors	BMJ	2002	30.31
2	551	Mundinger MO [31]	Primary care outcomes in patients treated by nurse practitioners or physicians: a randomized trial	JAMA	2000	45.54
3	323	Spitzer WO [32]	The Burlington randomized trial of the nurse practitioner	New England Journal of Medicine	1974	74.7
4	267	Venning P [33]	Randomised controlled trial comparing cost effectiveness of general practitioners and nurse practitioners in primary care	BMJ	2000	30.31
5	240	Bryant-Lukosius D [34]	Advanced practice nursing roles: development, implementation and evaluation	Journal of Advanced Nursing	2004	2.56
6	239	Kinnersley P [35]	Randomised controlled trial of nurse practitioner versus general practitioner care for patients requesting "same day" consultations in primary care	BMJ	2000	30.31
7	238	Newhouse RR [36]	Advanced practice nurse outcomes 1990-2008: a systematic review	Nursing Economics	2011	0.82
8	221	Melnyk BM [37]	The establishment of evidence-based practice competencies for practicing registered nurses and advanced practice nurses in real-world clinical settings: proficiencies to improve healthcare quality, reliability, patient outcomes, and costs	Worldviews on Evidence-Based Nursing	2014	1.99
9	182	Naylor MD [38]	The role of nurse practitioners in reinventing primary care	Health Affairs	2010	5.33
10	172	Kleinpell RM [39]	Nurse practitioners and physician assistants in the intensive care unit: an evidence-based review	Critical Care Medicine	2008	7.41
11	169	Brown SA [40]	A meta-analysis of nurse practitioners and nurse midwives in primary care	Nursing Research	1995	1.88
12	168	Lenz ER [41]	Primary care outcomes in patients treated by nurse practitioners or physicians: two-year follow-up	Medical Care Research and Review	2004	3.21
13	157	Sakr M [42]	Care of minor injuries by emergency nurse practitioners or junior doctors: a randomised controlled trial	Lancet	1999	60.39
14	154	Bryant-Lukosius D [43]	A framework for the introduction and evaluation of advanced practice nursing roles	Journal of Advanced Nursing	2004	2.56
15	147	Sackett DL [44]	The Burlington randomized trial of the nurse practitioner: health outcomes of patients	Annals of Internal Medicine	1974	21.32
16	143	Pulcini J [45]	An international survey on advanced practice nursing education, practice, and regulation	Journal of Nursing Scholarship	2010	2.66
17	136	Sheer B [2]	The development of advanced nursing practice globally	Journal of Nursing Scholarship	2008	2.66
18	121	Sox HC [46]	Quality of patient care by nurse practitioners and physician’s assistants: a ten-year perspective	Annals of Internal Medicine	1979	21.32
19	119	Donelan K [47]	Perspectives of physicians and nurse practitioners on primary care practice	New England Journal of Medicine	2013	74.7
20	118	Daly WM [48]	Nursing roles and levels of practice: a framework for differentiating between elementary, specialist and advancing nursing practice	Journal of Clinical Nursing	2003	1.97
21	113	Hill J [49]	An evaluation of the effectiveness, safety and acceptability of a nurse practitioner in a rheumatology outpatient clinic	British Journal of Rheumatology^*^	1994	5.61
22	111	Silver HK [50]	A program to increase health care for children: the pediatric nurse practitioner program	Pediatrics	1967	5.36
23	109	Wilson IB [51]	Quality of HIV care provided by nurse practitioners, physician assistants, and physicians	Annals of Internal Medicine	2005	21.32
24	108	Moote M [52]	Physician assistant and nurse practitioner utilization in academic medical centers	American Journal of Medical Quality	2011	1.43
25	107	Stanik-Hutt J [53]	The quality and effectiveness of care provided by nurse practitioners	Journal for Nurse Practitioners	2013	1.27
26	107	Mundinger MO [54]	Advanced-practice nursing -- good medicine for physicians?	New England Journal of Medicine	1994	74.7
27	105	Cowan MJ [55]	The effect of a multidisciplinary hospitalist/physician and advanced practice nurse collaboration on hospital costs	Journal of Nursing Administration	2006	1.27
28	102	Kuo YF [56]	States with the least restrictive regulations experienced the largest increase in patients seen by nurse practitioners	Health Affairs	2013	5.33
29	97	Gardner G [57]	Making nursing work: breaking through the role confusion of advanced practice nursing	Journal of Advanced Nursing	2007	2.56
30	95	Silver HK [58]	The pediatric nurse-practitioner program: expanding the role of the nurse to provide increased health care for children	JAMA	1968	74.7
31	94	Dierick-van Daele, AT [59]	Nurse practitioners substituting for general practitioners: randomized controlled trial	Journal of Advanced Nursing	2009	2.56
32	94	Kane RL [60]	Effects of a geriatric nurse practitioner on process and outcome of nursing home care	American Journal of Public Health	1989	6.46
33	93	Charlton CR [61]	Nurse practitioners’ communication styles and their impact on patient outcomes: an integrated literature review	Journal of the American Academy of Nurse Practitioners^**^	2008	1.42
34	92	Allen JK [62]	Community Outreach and Cardiovascular Health (COACH) trial: a randomized, controlled trial of nurse practitioner/community health worker cardiovascular disease risk reduction in urban community health centers	Circulation: Cardiovascular Quality and Outcomes	2011	5.07
35	91	Lowe G [63]	Time to clarify - the value of advanced practice nursing roles in health care	Journal of Advanced Nursing	2012	2.56
36	91	Makowsky MJ [64]	Collaboration between pharmacists, physicians and nurse practitioners: a qualitative investigation of working relationships in the inpatient medical setting	Journal of Interprofessional Care	2009	1.73
37	89	Buerhaus PI [65]	Practice characteristics of primary care nurse practitioners and physicians	Nursing Outlook	2015	2.83
38	88	Barlow SE [66]	Treatment of child and adolescent obesity: reports from pediatricians, pediatric nurse practitioners, and registered dietitians	Pediatrics	2002	5.36
39	86	Van Soeren M [67]	The role of nurse practitioners in hospital settings: implications for interprofessional practice	Journal of Interprofessional Care	2011	1.73
40	84	Swan M [68]	Quality of primary care by advanced practice nurses: a systematic review	International Journal for Quality in Health Care	2015	1.93
41	84	Jennings N [69]	The impact of nurse practitioner services on cost, quality of care, satisfaction and waiting times in the emergency department: a systematic review	International Journal of Nursing Studies	2015	3.78
42	84	Bauer JC [70]	Nurse practitioners as an underutilized resource for health reform: evidence-based demonstrations of cost-effectiveness	Journal of the American Academy of Nurse Practitioners	2010	1.42
43	84	Hooker RS [71]	Use of physician assistants and nurse practitioners in primary care, 1995-1999	Health Affairs	2001	5.33
44	83	Laurant MG [72]	An overview of patients’ preference for, and satisfaction with, care provided by general practitioners and nurse practitioners	Journal of Clinical Nursing	2008	1.97
45	82	Duffield C [73]	Advanced nursing practice: a global perspective	Collegian	2009	1.83
46	81	Manley K [74]	A conceptual framework for advanced practice: an action research project operationalizing an advanced practitioner/consultant nurse role	Journal of Clinical Nursing	1997	1.97
47	81	Spisso J [75]	Improved quality of care and reduction of housestaff workload using trauma nurse practitioners	Journal of Trauma - Injury, Infection and Critical Care^***^	1990	3.38
48	80	Dill MJ [76]	Survey shows consumers open to a greater role for physician assistants and nurse practitioners	Health Affairs	2013	5.33
49	78	Ohman-Strickland PA [77]	Quality of diabetes care in family medicine practices: influence of nurse-practitioners and physician’s assistants	Annals of Family Medicine	2008	4.69
50	78	Cogdill KW [78]	Information needs and information seeking in primary care: a study of nurse practitioners	Journal of the Medical Library Association	2003	2.04

Scopus Search

Our comparative Scopus search yielded some similar results to our WoS findings (The full Scopus list and citation frequencies are available upon request from the authors). The most highly cited paper was again Horrocks et al. [30] (767 citations), followed by Mundinger et al. [31] (636 citations). Thirty-two papers occurred in both datasets, albeit with different citation numbers.

However, despite using the same search terms in Scopus, 12 WoS papers with “advanced practice nurses” in the title did not appear within the Scopus top 50. The Scopus 50 ranked also included 18 separate and distinct publications that met our inclusion criteria yet did not appear in the WoS 50 dataset.

Comparison to Recent NP Bibliometric Studies

Jennings and Tori [22] conducted a bibliometric study of NP publications during 2007-2021 using Scopus, with publications being downloaded in September 2021. The top 20 NP papers, ranked by citation frequency, were listed. In comparison to our top 20, their second-ranked publication, Donelan et al. [47], had 133 citations and was ranked as number 19 in our WoS dataset. Compared to their published top 20, nine publications were in our WoS listing as well [52,53,56,59,62,64,72,76,77].

In Huang et al. [21], four publications in their published top 10 most cited were duplicated in our top 10, albeit with different ranking and number of citations [30,36,38,39]. Their publications were accessed in June 2019 via WoS.

Publication Dates

The publication dates spanned six decades (1960-2015) (Table 2). The oldest article was published in 1967 and ranked 22 with 111 citations [50]. The first citation of this publication was in 1967, and the most recent citation was in 2020, 53 years after its initial publication. The greatest density of highly cited articles was published in the decades 2000-2010 (n = 22; 44%) and 2010-2020 (n = 16; 32%), followed by the 1990s (n = 6; 12%), the 1970s (n = 3; 6%), the 1960s (n = 2; 4%), and the least published in the 1980s with only one publication (n = 1, 2%). For a single year, the most articles (n = 5) were published in 2008.

**Table 2:** Publication dates

Decade	Number of publications
1960s	2
1970s	3
1980s	1
1990s	6
2000s	22
2010s	16

Country of Origin

The country of origin was determined according to the first author. A majority of the top 50 papers originated in the United States (n = 31; 65%), with the United Kingdom (n = 7; 14%) and Canada (n = 6; 12%) following. Australia (n = 4; 8%) and the Netherlands (n = 2; 4%) were also represented.

Authors

A total of 194 authors contributed to the publications, and 32 authors contributed to more than one. It was not possible to determine who the “lead” was for each publication; an assumption was made that the first author was the primary contributor. There were 47 first authors.

Three contributors were listed as first authors for two articles: Mary O’Neil Mundinger was listed as first author for two publications [31,54] and as a second author [41]. Her publications were a randomized controlled trial (RCT) in patients treated by NPs and physicians and the follow-up to the said RCT. Mundinger was the sole author of the publication Advanced-practice nursing -- good medicine for physicians?. Denise Bryant-Lukosius, as the first author, published two articles, both with Alba DiCenso [34,43]. These publications discussed aspects of the implementation and evaluation of advanced practice roles. There was sole authorship for five publications. The most multi-authored publication had 12 authors [62].

Authors’ Discipline

The discipline of the 47 first authors was determined by a review of credentials. Of those authors for whom credentials/disciplines were not listed in the publication but whose email addresses were listed, three authors confirmed his/her or their disciplines directly [30,35,38,56]. One email address was undeliverable and, as a result, unable to confirm the discipline of the first author [53]. One paper did not provide an email address and we were unable to confirm the discipline of the first author [49].

For the first author, a nursing discipline was the most prevalent (n = 22; 47%), followed by physicians (n = 9; 19%), “other” (n = 7; 15%), NPs (n = 5; 11%), unknown (n = 2; 4%), and physician assistants (n = 2; 4%). Multidisciplinary authorship with nurses, NPs, and physicians is noted in 21 publications (40%). The first top three ranked publications were authored by many different disciplines, including nurses and physicians, but no NPs were identified. It is only at the fifth-ranked publication that an NP is identified as collaborating as the fourth and last author [34].

Institutions

Institutions identified were those affiliated with the first author of the publication. There were 38 institutions noted, with eight institutions associated with more than one article (Table 3). The greatest number of papers (n = 5) were from McMaster University in Ontario, Canada.

**Table 3:** Institutions

Institution	Number of publications
McMaster University (Canada)	5
Columbia University (United States)	3
University of Texas (United States)	3
John Hopkins University (United States)	2
Queensland University of Technology (Australia)	2
The Ohio State University (United States)	2
University of California (United States)	2
University of Colorado (United States)	2

Publication Types

Almost one-third (n = 14; 28%) of the publication types were classified as qualitative, followed by systematic reviews/meta-analyses (n = 10; 20%) and clinical-experimental-randomized (n = 10; 20%). This was followed by discussion papers (n = 8; 16%) and observational cross-sectional (n = 5; 10%), with one literature review, editorial, and observational cohort.

Topics

The most common topic area in our dataset was the impact of the role (n = 29, 58%). Of these 29 publications, 19 (66%) discussed the direct comparison of NP impact in comparison to physicians. Also, within the topic of impact, seven publications examined both the NP and PA together, with no distinction between these roles [39,46,51,52,71,76,77].

The second most common topic was role development (n = 12, 24%), followed by registration/licensing (n = 6; 12%) and scope of practice (n = 2, 4%). One publication, the editorial by Mundinger, discusses many aspects of the impact of the NP role but also scope, regulation, and role development [54].

Journal Characteristics

The 50 publications were represented across 30 journals. The journal with the most publications was the Journal of Advanced Nursing (n = 5, 10%), followed by Health Affairs (n = 4, 8%), New England Journal of Medicine (n = 3, 6%), the BMJ (n = 3, 6%), Annals of Internal Medicine (n = 3, 6%), and the Journal of Clinical Nursing (n = 3, 6%).

The journal with the highest IF was the New England Journal of Medicine (74.7) and the lowest was Nursing Economics (0.82). Most articles (n = 35; 70%) were published by journals with an IF of greater than 2. Of the 30 journals listed, 63% (19) had IFs greater than 2.0 and were mostly related to medicine (combined medicine, critical care medicine, cardiac, pediatrics, and rheumatology; n = 11; 58%). There were four nursing journals with an IF greater than 2. The highest IF nursing journal (IF = 3.78) is the International Journal of Nursing Studies, with one publication that ranked at #41. Of the journals that had an IF of less than 2 (n = 11; 37%), the majority were nursing journals (n = 8; 73%), and the remaining were in the Health Care Sciences and Services journal category (n = 3; 27%).

Discussion

In this study, we conducted a bibliometric review of the top 50 cited publications related to NPs/APNs using the WoS database and a supplementary Scopus search. This analysis provides a glimpse into the articles that have influenced the development and history of the NP movement.

Citation Frequency

Using the metric of “high” citation counts in the WoS database allowed us to compile a listing of the top 50 publications that could be considered influential to the NP profession. This process provides an opportunity to recognize these impactful papers, and we propose that these papers have contributed to influencing the development and evolution of the NP domain globally.

It has been suggested that publications within the nursing field receiving 150 citations or more could be considered “exceptional papers” [11]. It is noteworthy that 28% of articles in our dataset were cited at least 150 times. These authors further propose that papers cited more than 50 times are considered “very good” and those with more than 100 citations are “excellent”. Based on these parameters, an additional 28% of our publications can be deemed to be papers of excellence, and the remaining 44% are considered “very good”. This could further suggest that all the papers in the “top 50” have made some impact on modern-day NP practice. Varghese et al. suggest that within a small specialty, articles that are cited more than 100 times could be considered a “citation classic” [24]. Denoting an article as a citation classic implies that it likely created transformation with a field, causing shifts in understanding and change.

The most highly cited paper in our WoS and Scopus lists was ranked third in Huang et al. [21], The Systematic review of nurse practitioners working in primary care can provide equivalent care to doctors from the United Kingdom, published in the BMJ [30]. This citation classic, with 656 citations in WoS (Scopus: 767 citations), has persisted with its use/influence over the last 19 years. This publication involved the systematic review of 34 papers, comprised of 11 RCTs and 23 observational studies comparing NP care to physician care of patients in primary care settings. Outcomes in this study demonstrated that although NPs had longer consultations and ordered more investigations, patients were more satisfied with their care, with no differences in health status, and with “quality of care was in some ways better for nurse practitioner consultations” [30]. It is interesting to note the recent citation of this publication was in the observational study in Italy by Bezze et al. [80]. Italy is in the early stages of the APN/NP role, but the actual implementation is not evident. Horrocks et al. [30] were cited within the Bezze et al. article twice, noting that Italian pediatric nurses, given the appropriate educational support and experience, can provide enhanced, appropriate healthcare services to children [80]. The findings called for increased investigation of the activities of nurses working in pediatric practices and underlined the potential of developing the nursing profession at the community level to enable more autonomy and independence. This one, recent citation of the Horrocks article is an example of the publication perhaps influencing advanced practice of nurses in a country where the profession is immature.

Mundinger et al. was the second highest cited article in both WoS (551 citations) and Scopus (635) [31]. This American RCT compared the effectiveness of NP care with the “usual care” of primary care physicians as the “control”. The NP had the same practice characteristics, such as authority and responsibilities, as the doctors. Outcomes demonstrated that patients under the NP care had no differences in patient-reported health status, a minimal but attributable small difference in patient satisfaction and that the NP group had a lesser but statistically significant difference in decreased diastolic blood pressure in patients with hypertension. Strengths of this study were the sample size The third-ranked publication in our WoS list, The Burlington randomized control trial of the nurse practitioner, also appeared in the Scopus list but with fewer citations (300) [32]. The article also demonstrates longevity in its performance, as it was cited 45 years after its publication in Maier [81]. This study, using surveys sent to 39 European countries, as well as the United States, Australia, New Zealand, and Canada, aimed to assess the status and regulations regarding the prescriptive authority of nurses, which tends to be a specific scope of practice domain for NPs [81]. The Spitzer et al. publication was used to augment the long tradition of nurses working in advanced practice roles, including the practice of prescribing medications [32].

The Burlington randomized trial occurred in Ontario, Canada, and involved comparing health care outcomes such as physical functionality and patient satisfaction when substituting NPs with the usual care provided by family physicians [32]. Like Horrocks et al. [30] and Mundinger et al. [31], healthcare outcomes were equal and patient satisfaction and professional satisfaction were favorable. However, it was acknowledged that this novel delivery of primary health care was not financially advantageous to physicians in the province. In our dataset, this publication is the earliest RCT assessing the impact of NP practice in comparison to physicians. We surmise that this may be the first published RCT to compare NP to physician care. We also consider this paper to be a classic publication/landmark study, as further evidenced by the reprinting of the paper in the Journal of the American Academy of Nurse Practitioners in 1990: A classic manuscript reprinted in celebration of 25 years of progress [82].

Scopus Search

Our concurrent Scopus search for top publications using the same search terms highlighted differences uncovered by the database chosen. Our WoS analysis of the ranking, prolific authors, topics, and publications, for example, yielded different results. However, it is important to acknowledge that there are many publications appearing in both databases, and the Scopus listings added further NP publications. Combining the two lists for future analysis could enable a more robust and comprehensive collection of pivotal NP publications.

Comparison to Recent NP Bibliometric Studies

Despite having a similar research topic and methodology, other publications found different citation rates [21,22]. This is not surprising given the different databases used, search strategy parameters, and years of publications searched. Citation rates are not static numbers and fluctuate, and the dates of publications accessed were different for all three studies. However, there are duplicate studies in each, providing some concordance about influential and noteworthy NP publications.

Publication Dates

The literature comprising the WoS dataset has a lengthy history, spanning back to the 1960s. In CA, the date of publication is thought to have some influence over the citation rate. Older publications have had the time to be cited more often than more recent publications, and the number of citations increases with time, and time passed is necessary to accrue citations [5,9,16].

The oldest publication, a citation classic by Silver et al. [50], details the educational training necessary to prepare nurses to become pediatric NPs and continues to be cited an impressive 53 years after its publication date [83]. In the editorial by Curry [83], she challenges persistent notions regarding NPs. One assumption she attempts to defy is that NPs are simply a solution to “physician shortages” and “gap fillers”. She describes how Silver et al. [50] in their “seminal article” were very clear that the intention of the pediatric NP role was to help meet the increasing need for improvement in access to care, not as “physician stand-ins”. Curran also notes that the authors highlighted that collaboration as a team was ideal and that nursing brings a different perspective to the care provided to children. The article by Silver, Ford, and Stearly continues to be impactful in influencing and supporting NP practice many years after its publication and is used to assuage misconceptions about the role.

It is critical to highlight that the Silver et al. [50] publication was not captured in the Scopus database of 50 and a missed opportunity to highlight this work would have arisen if we confined our search to Scopus. Although Scopus may index more journals than WoS, Scopus may have had limited access to publications before the 1990s [84].

Of our most recent publications, three were published in 2015, with Buerhaus et al. [65] having the most citations and were available online in August 2014. This study involved comparing the demographics and practice characteristics of 467 primary care NPs to 505 primary care physicians in the United States. The differences were related to practice settings, populations served, and NPs working fewer hours and seeing fewer patients. Issues were noted related to government regulations hindering NPs’ abilities to provide autonomous care, such as admitting patients and working within the hospital setting. However, both groups of primary care providers indicated that increasing the supply of NPs would result in better team collaboration and practice, with resultant improved access, especially for more vulnerable patients.

The increase in articles published in the decades 2000 to 2015 coincides with the advances in the profession and development of education programs. The International Council of Nursing (ICN) noted that after progression in the United States and Canada in the 1960s/1970s and the United Kingdom in the late 1980s, the concept of the NP rose internationally after the 2000s [1].

Country of Origin

The top countries featured in our dataset (United States, United Kingdom, Canada, and Australia) also reflect previous CA results [21,22]. The ICN also highlights these four countries as ones that have established distinct NP roles in their healthcare systems [1].

Authors’ Discipline

We used the “first author” as a heuristic to identify the “top author” in order to isolate and highlight researchers based on the number of absolute citations and number of papers. Our review provides a listing of researchers, authors, and clinicians who have produced literature that can be considered impactful to the profession. The minimum number of citations received by a publication was 78, and publications with 30-50 citations are considered a productive output [18]. This CA has demonstrated all the authors could be considered “prolific” concerning advanced practice nursing.

There were five publications with sole authorship. Generally, multi-authored articles are cited more frequently [27]. This inclination towards multi-authorship echoes the importance of collaboration, not only in research endeavors but also in terms of involving different roles in a multidisciplinary team. In turn, effective collaboration between nurses and physicians has been linked to improvements in patient care [50].

Along with multi-authorship, we also saw evidence of a multidisciplinary approach to the publications. Although nursing dominated as the most common discipline assigned to the first author, 40% of the publications were authored by the collaborative efforts of nurses, physicians, NPs, and others. Research is moving in a direction that lends itself to becoming more multidisciplinary, and by utilizing a variety of skill sets and perspectives, a more robust depiction of research solutions and insights can be obtained. The value of integrating medicine and nursing continues to be vital to providing innovative solutions for the complexities of healthcare and ensuring adequate healthcare services.

While it is evident that collaboration in research can be beneficial, NPs as lead authors or researchers should be encouraged. An NP was the lead author identified in only five of the 50 (10%) publications. The unique perspective and role of the NP is ideally positioned to lead research by identifying issues of clinical relevance from their own practice focus and observations.

Publication Types

The publication types were evenly represented between qualitative and quantitative designs. Qualitative research related to NPs provides a perspective to help understand and interpret the meaning surrounding the advanced nursing practice paradigm [14]. Many of the top 10 ranked publications were either RCTs or systematic reviews/meta-analyses and were cited most frequently. The rationale for this could be that RCTs are widely regarded as the “gold standard” for evidence generation. Consequently, these studies are often published in journals with higher IFs, which, in turn, can influence their citation counts [8,23]. The type of publications that made the highly cited list is influenced by the database we chose to extract our publications from. We did not critique or review the quality of each publication independently, but this review provides an awareness of the types of articles and methodologies.

Topic

Almost 60% of the publications focused on the topic of the impact of the NP. Within these publications, the NP’s influence and outcomes were discussed related to safety, effectiveness, quality of care, and cost-effectiveness. Many publications discussed that the need for NPs arose due to physician shortages, a crisis related to the accessibility of care for certain populations, as well as strides to provide more collaborative and comprehensive management to patients. The aim of many studies related to the impact of the NP topic area involved whether NPs could provide care that was previously in the exclusive domain of physicians and in a safe manner with comparable outcomes. The top three most highly cited papers in our WoS all focused on the impact, safety, and effectiveness of NPs compared to physicians. Other studies also explored impact. For example, Newhouse’s et al. systematic review involved 14 RCTs and 23 observational studies from 1990 to 2008, comparing the care of NPs to care managed by physicians [36]. Health outcomes such as functional status, glucose control, lipid control, and blood pressure were examined, as well as patient satisfaction. Emergency Department usage, hospitalizations, and “length of stays” were also evaluated. Generally, their findings indicated that NPs provided similar and/or sometimes more favorable outcomes than exclusive physician care. There are other practice settings that discuss impact, such as the study by Sakr et al. publication from the United Kingdom that compared the care provided by Emergency Room NPs to junior doctors for minor injuries [42]. Their findings revealed that NPs “were better at” documenting histories and had comparable skills in examinations, treatments, follow-up, and interpretation of X-rays. A Dutch study investigated outcomes for NPs substituting for general practitioners [59]. They concluded that NPs provided comparable medical care but had more follow-up visits, and longer appointments. 

These publications and our developed topic areas were not evaluated for their study design structure or validity, as this review was an exploratory bibliometric scan and not a systematic review. However, future researchers might wish to build upon our preliminary work and extend the analysis related to topics using mapping methods or thematic analyses.

Journal Characteristics

Of the 30 journals represented in our sample, 12 are nursing-specific, accounting for 20 articles published. Applying Hack’s et al. criteria [11], five of those 20 articles had citations greater than 150 times, alluding to “exceptional”; five articles had more than 100 citations, deemed “excellent”; and the remaining 10 articles had citation numbers less than 100 but greater than 50, indicating “very good” papers. These findings contrast with other studies in nursing journals. Martin-Del-Rio et al. [23] examined “stress” in nursing journals: the citation numbers “low in comparison to other fields of study” were mostly cited less than 100 times. Studies have found citation numbers of articles in nursing journals were considered low in contrast to other journal categories, such as medicine [85].

There are two nursing-focused journals with the most publications: the Journal of Advanced Nursing and the Journal of Clinical Nursing. The Journal of Advanced Nursing had the most articles published (n = 5), with an IF of 2.6 and a range of number of citations from 91 to 240. Similarly, Giménez‐Espert and Prado-Gascó showed the Journal of Advanced Nursing had the greatest number of publications and citations of all other nursing journals analyzed [86].

Health Affairs had the second most publications (n = 4); it is focused on international health policy issues with exposure to a broad audience. The remainder of the journals were in the categories of combined medicine-focused categories (n = 11) and healthcare sciences and services/public health/library Science (n = 7).

Nursing intersects other disciplines, and this intersectionality is reflected in the diversity of journals hosting our publications. This is indicative of the multidisciplinary nature of NP practice as well as how the role influences wider health policy. 

IF

The IF is used to provide an “objective” metric of journal quality. Those possessing higher IFs are thought to be more influential [27]. However, IF rankings cannot be interpreted in isolation as they are subject to much criticism, controversy, limitations, and inherent “dangers” if applied erroneously [18,27,85,87].

There is a diversity of journals in our dataset with a wide range of IF values. The top four ranked publications in our WoS 50 were in medical journals with high IFs. These journals could be considered eminent medical journals, which typically have high IFs [64]. It is also suggested that well-known journals have a large readership audience, which itself affects the IF [17]. In contrast to medical journals, nursing journals tend to have lower IFs [85,88]. There may be an inclination to compare the IF of medical journals and citations with the IF of nursing journals and associated citations, but this cross-subject comparison is fraught with limitations [85]. Despite nursing journals’ inclination to have lower IFs, this does not negate their worth or importance in providing a medium to circulate information to the right audience.

Jia et al. [10] and Li et al. [13] discuss studies demonstrating journal IF being strongly related to the number of citations of a publication. The first four publications in our ranked list were from medical journals with high IFs. Conversely, there are articles from “lower” IF nursing journals in our top 10 ranked articles. For example, Nursing Economics has the lowest IF (0.82) of all our journals yet has a publication with 238 citations and is ranked seventh on our list [36]. We did not explore a possible association between the IF of a journal and the ranking achieved on citation numbers or the number of articles per journal with a high IF.

Limitations

There are intrinsic shortcomings in CA, which cannot be the sole arbiter of a publication’s worth. The lack of many citations of a publication does not mean it is not being read or influential. Citation data changes substantially with time. The citations in and of themselves do not distinguish whether a paper was cited positively or in a negative manner, such as refuting the paper’s credibility. Citation numbers may be affected by the journal the article is published, and we did not correct for self-citations. Publications that are older have more time to be read and communicated, so the number of citations may benefit older publications while newer, less cited publications may be overlooked. 

A limitation exists with having one author (A.H.) responsible for the data collection, and this also made it necessary to limit our sample size to 50 publications. We did not go into more depth with CA such as citation density, tracking trends, or the h-index of the authors. Nor did we weight the ranking of the list based on the IF of the journal in which the article was published.

The database chosen greatly influences the publications and citation counts, as evidenced by the material differences in our datasets for WoS and Scopus. The three different databases: WoS, Scopus, and Google Scholar all have unique aspects, including access reach. The WoS accesses articles from the 1900s, whereas Scopus includes journals from the 1990s, and Google Scholar focuses on digital materials from the mid-1990s [14,27]. WoS includes only the English language; therefore, we are potentially missing literature from other languages [23]. Both Scopus and WoS have access to different sets of scholarly journals, with Scopus indexing more journals than WoS [84]. Google Scholar indexes a variety of literature, including gray papers and many items published on the internet, is less specific in utilizing peer-reviewed publications, and tends to have more citations than the other two databases [5,9,84].

The search terminology and title search restricted our results to Nurse Practitioners OR Advanced Practice Nursing. This could have caused us to overlook some relevant studies. It is interesting to note that within our dataset of publications, there were many different terms within the text of the article to describe the NP or APN.

Future studies on CA of the NP literature would benefit from using a combination approach of all three databases.

## Conclusions

The birth, development, and maturation of a profession is complex and multifactorial. It is impossible to implicitly define what literature influences and shapes a field of study. However, using CA and applying a judicious lens, we have gathered what can be considered a “canon.” Our review has provided an organized means of identifying sentinel classics that have influenced the NP profession internationally. The findings of this study provide a means for readers to become familiar with some of the top publications, authors, and journals that have made contributions toward the NP as a profession.
